# Visualization of the microstructure and distribution of follicles in deep human ovaries using speckle-modulated optical coherence microscopy

**DOI:** 10.1371/journal.pone.0331638

**Published:** 2026-07-08

**Authors:** Koichiro Ito, Momoko Kanaya, Seido Takae, Nao Suzuki, Kosuke Tsukada

**Affiliations:** 1 Center for Applied Physics and Physico-Informatics, Graduate School of Mathematical and Physical Sciences, Keio University, Yokohama, Japan; 2 Department of Obstetrics and Gynecology, St. Marianna University School of Medicine, Kawasaki, Japan; Karpagam University: Karpagam Academy of Higher Education, INDIA

## Abstract

Ovarian tissue cryopreservation and transplantation remain important fertility preservation options for children, adolescents, and young adults requiring immediate treatment for their primary disease. Reliable visualization of follicle-containing regions may provide useful information for future ovarian tissue assessment. Despite its potential as a noninvasive follicle visualization method, optical coherence microscopy (OCM) remains limited by spatial resolution, imaging depth, and speckle noise when resolving fine follicular structures in deep ovarian tissue. In this study, we applied speckle modulation to a near-infrared wavelength-swept OCM system to visualize follicular microstructures in deep ovarian tissue. OCM images of 4-day-old mouse ovaries revealed dense superficial distributions of primordial follicles, which were distinguishable from primary follicles surrounded by a single layer of granulosa cells. Secondary follicles were predominantly observed in 14-day-old mouse ovarian tissue, and their internal microstructures were visualized. The proposed OCM approach visualized primordial follicular microstructures to depths of approximately 300 and 500 μm in ovarian tissues from a 15-year-old patient with acute myeloid leukemia and a 14-year-old patient with acute lymphoblastic leukemia, respectively. In the examined specimens, follicle localization varied with imaging location and depth, and similar structural features were also observed in HE-stained sections. Although challenges remain regarding imaging depth, acquisition time, and safety validation, these findings demonstrate the technical feasibility of visualizing follicular microstructures in human ovarian tissue and support future studies on ovarian tissue assessment.

## 1. Introduction

Radiation and chemotherapy for cancer treatment in children, adolescents, and young adults (CAYA) often result in ovarian dysfunction or failure [1,2]. The decline in childhood cancer mortality in recent years has increased interest in post-remission quality of life [3]. In this context, ovarian tissue cryopreservation (OTC) is emerging as a promising fertility preservation (FP) therapy for the next generation.

In OTC, ovarian tissue is collected before gonadotoxic therapy, cryopreserved, and subsequently autotransplanted when pregnancy is desired [4–7]. Since the initial report in 2004 [8], the number of reported cases of ovarian tissue transplantation (OTT) has steadily increased, with 130 cases reported in 2017 [9], and estimated to approach 200 by 2020 [10]. Most reported OTTs result in adequate restoration of ovarian function [11,12]. The functional lifespan of transplanted ovarian tissue depends on its primordial follicle count [13]. Furthermore, follicles within ovarian tissue exhibit a heterogeneous distribution, with primordial follicle density varying by two or more orders of magnitude within a single cortical section [14]. Consequently, the primordial follicle density of transplanted ovarian tissue governs transplantation efficacy. Therefore, establishing a noninvasive method to visualize follicle localization and microstructure may provide useful information for future ovarian tissue assessment.

Follicles consist of oocytes surrounded by granulosa and theca cell layers. As follicles mature, their microstructure undergoes characteristic changes. For instance, the granulosa cells surrounding the oocyte exhibit a flattened morphology in primordial follicles, transition to a cuboidal configuration in primary follicles, and subsequently mature into multilayered secondary follicles. Primordial follicles, which have an approximate diameter of 20–30 μm, are relatively abundant in the ovarian cortex at a depth of 100–1,000 µm [15]. Therefore, visualizing follicular microstructure, identifying maturation stages, and assessing follicle localization require both sufficient imaging depth to cover the regions in which follicles are frequently present and a spatial resolution of approximately 10 μm.

Optical coherence tomography (OCT) is a noninvasive imaging technique that visualizes the internal structures of tissues to a depth of several millimeters [16]. This technique is widely used in ophthalmology [17,18] and dentistry [19,20] as well as to visualize the gastrointestinal tract [21,22], coronary vessels [23,24], the colon [25], and the breast [26]. Recent studies have also utilized OCT for ovarian tissue imaging [27–29]. For example, primordial follicles in mouse, bovine, and human ovaries have been visualized using full-field OCT (FF-OCT) with a spatial resolution of approximately 1 µm [30–32]. We previously demonstrated the feasibility of evaluating primordial follicle distribution and ovarian reserve in human ovaries [31]. However, the limited imaging depth (~100 µm from the tissue surface) and speckle noise, which obscures follicular microstructure, limit its application to ovarian tissue assessment.

Speckle noise reduction methods include optical approaches, such as angular compounding [33], averaging algorithms [34], frequency compounding [35], and polarization diversity [36]. Image-processing approaches, including adaptive filters and wavelet analysis [37–42], and machine-learning techniques [43–47] can also reduce noise in OCT images. Although several methods for noise reduction have been established for OCT images of the retina [34,48,49], comparable methods suitable for ovarian tissue, which possesses a more heterogeneous structure than the retina, remain unestablished. While Gaussian filters have effectively reduced noise in segmented OCT images of mouse ovaries [50], filtering generally degrades spatial resolution. Therefore, accurately restoring noise-degraded structural information without compromising resolution remains a fundamental challenge.

Recently, Liba et al. proposed speckle modulation, which enables the creation of an unlimited number of uncorrelated speckle patterns by moving a diffuser plate within the optical path, thereby demonstrating that speckle noise can be effectively reduced without degrading resolution [51]. Beyond preserving resolution and reducing noise, the simplicity of implementing speckle modulation in OCT systems makes it suitable for visualizing follicular microstructure.

In this study, we present an optical coherence microscopy (OCM) system capable of visualizing follicular microstructure across different maturation stages at depths with high follicle density. Specifically, applying phase shift–based speckle noise reduction processing to an optical system based on a 1,300-nm wavelength-swept OCM enabled the visualization of follicular microstructures. We first evaluated whether speckle-modulated OCM could visualize follicular microstructures across different maturation stages in mouse ovaries and then investigated the feasibility of applying this approach to human ovarian tissue.

## 2. Materials and methods

### 2.1. OCM system setup

**Fig 1:**
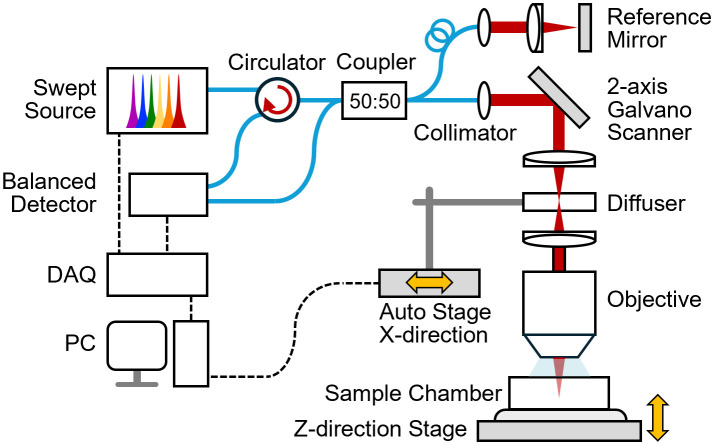
Schematic of the OCM optical system. Laser light from the sample arm is reflected by a galvanometer scanner and focused onto the ovary via a diffuser used for speckle modulation and an objective lens.

Fig 1 shows the OCM system for noninvasive visualization of follicular microstructure. A swept-source laser (HSL2100, Santec) with a 1,310 nm center wavelength, a 171 nm bandwidth, and a 20 kHz frequency served as the light source. Laser light passing through a circulator (CIR1310-APC, Thorlabs) was split into reference and sample arms in a 50:50 ratio using a fiber coupler (TW1300R5A2, Thorlabs). The reference light was reflected by a mirror via a polarization controller. The sample light was focused onto the sample via a galvanometer scanner, a diffuser plate (DG10–600, Thorlabs) mounted on a linear motion stage, and a 20 × water-immersion objective lens (XLUMPLFLN20XW, Olympus) with a numerical aperture of 1.0. A differential amplifier (PDB570C, Thorlabs) detected the interference signal between the sample backscatter and the reference mirror reflection. Data acquisition and processing were performed using a DAQ board (HAD-5200B-S, Santec) and original software.

A USAF 1951 contrast resolution target was placed on the focal plane of the objective lens, and the lateral spatial resolution was evaluated from the resulting OCM images. Depth resolution was evaluated as the full width at half maximum (FWHM) of the point spread function (PSF), averaged over 20 A-scans acquired with a mirror positioned at the focal plane.

### 2.2. Preparation of the ovarian phantom

To prepare an ovarian phantom containing glass beads embedded in a polyacrylamide gel, a 4.9% polyacrylamide gel was mixed with 5% Intralipid as a scattering agent [52]. Next, glass beads with a diameter of 30 ± 2 µm (SPL-30, UNITIKA), mimicking ovarian follicles, were suspended and gelified. The phantom was then placed in a custom-designed sample holder filled with double-distilled water.

### 2.3. Mouse and human ovarian tissue samples

Animal experiments were performed according to protocols approved by the Animal Experiment Committee of the Graduate School of Medicine, St. Marianna University (protocol nos. #2304008, #2304009). Ovarian tissues were collected from female mice at 4 days, 14 days, and 20–24 weeks of age after sacrifice via cervical dislocation and were immediately placed on ice. The average body weights of the mice were 3.4 g (day 4, n = 3), 7.9 g (day 14, n = 3), and 39.7 g (week 20–24, n = 3), respectively. Within 12 h of collection, the tissue was placed in a sample chamber filled with phosphate-buffered saline, and OCM images were acquired.

Human tissue experiments were approved by the Clinical Trials Subcommittee of the Institutional Review Board for Biomedical Research at St. Marianna University School of Medicine (Approval No. 6272, IN000023141). The recruitment period for this study spanned January 5, 2024, to March 31, 2026. The details of this clinical study were explained to all patients or their families by their study investigators, and written informed consent was obtained from all participants or their representatives. This study was conducted in accordance with applicable ethical guidelines, including the Declaration of Helsinki and the Act on the Protection of Personal Information. Human ovarian tissue samples were obtained from a 15-year-old female who developed acute myeloid leukemia (AML) secondary to familial thrombocytopenia and a 14-year-old female diagnosed with acute lymphoblastic leukemia (ALL). The cryopreserved ovarian tissue, approximately 10 mm × 10 mm × 1 mm thick, was thawed and cut into four equal pieces, each approximately 5 mm × 5 mm square. The tissue was placed in a sample chamber filled with culture medium (G-MOPS™, Vitrolife, Gothenburg), and OCM imaging was performed.

### 2.4. Hematoxylin and eosin (H&E) staining

The ovarian tissues used for OCM imaging were fixed in 4% paraformaldehyde, embedded in paraffin wax, sectioned at a thickness of 5 μm, and stained with H&E following standard protocols.

### 2.5. Process for acquiring OCM images

The laser light was focused vertically onto the specimen through an objective lens to obtain OCM signals along the depth direction (A-scan). By repeatedly scanning the laser horizontally with a galvanometer mirror (B-scan), volume data with an area of 666 × 666 µm^2^ and a depth of 75 µm were acquired. In experiments using human ovarian tissue, five primary imaging locations were selected: the center of the approximately 5 × 5 mm^2^ tissue specimen and four additional positions located 2 mm above, below, to the left, and to the right of the center. Because the objective of this study was to evaluate the capability of visualizing follicular microstructures rather than estimating the representative follicle density distribution, additional regions containing morphologically identifiable follicles were also imaged for structural assessment. Moving the diffuser plate in the B-scan direction at a speed of 2.4 µm/s for 50 µm was repeated 20 times. Volume data were acquired each time and subsequently averaged. En-face images were generated from these data and smoothed with a Gaussian filter (standard deviation = 0.8). To compensate for the shallow depth of field of the high-numerical aperture objective lens, the lens was axially shifted in 30 µm increments to repeatedly acquire volumetric data. All data were then merged into a single three-dimensional (3D) image after removing any overlapping parts. 3D images of the phantoms and mouse ovarian tissues were generated using the open-source software 3D Slicer, with glass beads and oocytes marked as spheres.

## 3. Results

### 3.1. Evaluation of lateral and depth resolution

**Fig 2:**
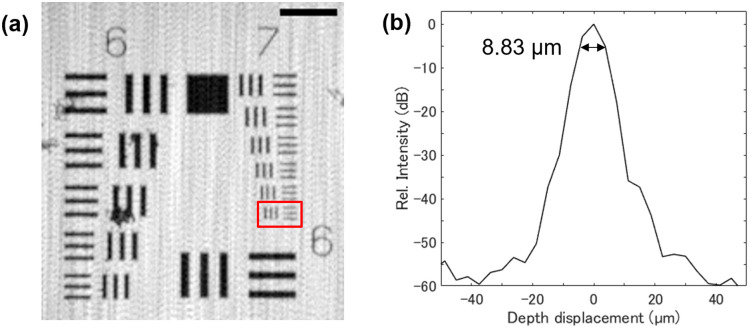
Evaluation of spatial resolution in the horizontal and axial directions. (a) OCM image of a 1951 USAF resolution test chart. The resolution indicated by the red frame is less than 4.38 µm. (b) Point spread function obtained in the depth direction by an A-scan. The depth resolution in air is 8.83 µm.

The OCM image of the 1951 USAF resolution test target shown in Fig 2(a) was spatially resolved down to the smallest element in the horizontal and vertical directions, indicating that the horizontal spatial resolution was < 4.38 µm. The theoretical value calculated from the FWHM of the wavelength spectrum of the light source was 5.78 µm in air. The discrepancy between the measured and theoretical values resulted from deviations of the wavelength spectrum from an ideal Gaussian profile. Fig 2(b) shows the PSF in the depth direction obtained by placing a mirror on the focal plane of the objective lens, from which the depth resolution in air was determined to be 8.83 µm. The *in vivo* spatial resolution, calculated using water’s refractive index (n = 1.33), was 3.29 µm laterally and 6.64 µm axially. This spatial resolution enables 3D visualization of primordial follicles with diameters of approximately 30 µm and the fine structures of secondary follicles.

### 3.2. Ovarian phantom imaging test

**Fig 3:**
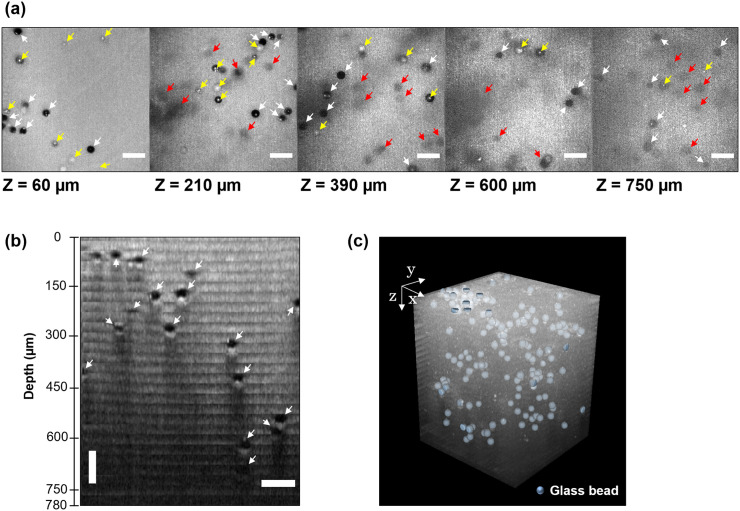
OCM images of an ovarian phantom. (a) En-face images at different depths (Z = 60–750 µm) from the surface. White arrows indicate actual beads present at the focal plane, and red arrows indicate shadows caused by signal attenuation due to beads in the upper layers. White spots indicated by yellow arrows represent light reflections caused by Fresnel reflection losses at the interface between the glass beads and the surrounding matrix. (b) Representative XZ cross-sectional (B-scan) image (see S1 Video in S1 File). (c) 3D diagram of the glass bead distribution (see S2 Video in S1 File). Scale bar = 100 µm.

Glass beads with diameters of 30 μm were visualized at a depth of up to 700 μm from the ovarian phantom surface (Fig 3(a)). The clear shadows of the black circles (white arrows) indicate the actual beads, whereas the blurred shadows (red arrows) indicate signal attenuation caused by the beads above. The yellow arrows indicate white spots within the dark shadows, which represent reflections caused by Fresnel reflection losses at the interface between the soda-lime glass beads (refractive index ≈ 1.52) and the surrounding aqueous matrix (refractive index ≈ 1.33). Fig 3(b) and S1 Video in S1 File display the cross-sectional images in the B-scan direction. In deeper regions, the signal contrast with the surrounding area decreased compared to the surface region due to light attenuation caused by scattering and absorption. Fig 3(c) and S2 Video in S1 File display the distribution of glass beads detected in a volume of 666 × 666 × 810 µm³. These results suggest the feasibility of visualizing 30 µm-diameter primordial follicle distributions within deeper ovarian regions.

### 3.3. Visualization of follicles in mouse ovaries

**Fig 4:**
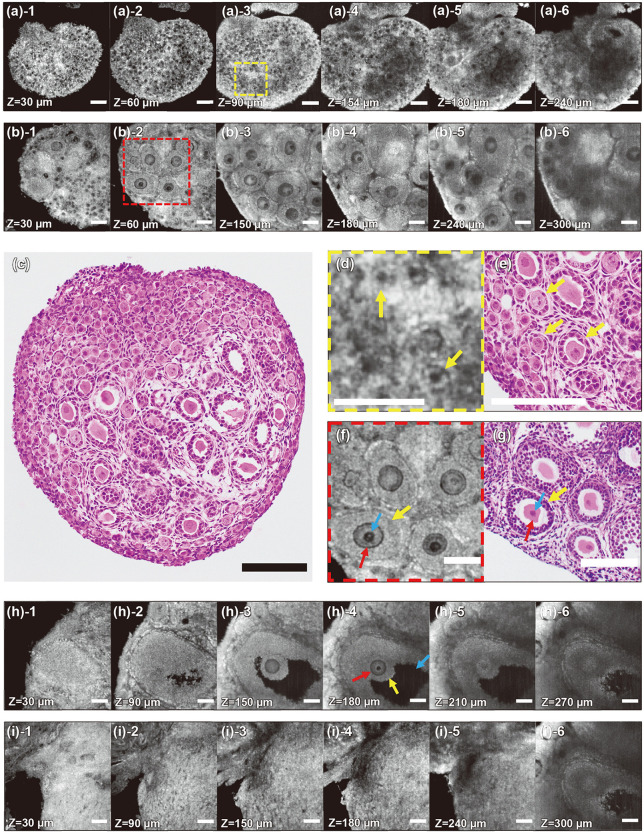
OCM images of mouse ovaries. (a and b) En-face images at depth Z from the tissue surface of (a) 4-day-old and (b) 14-day-old mice (see S3 and S4 Videos in S1 File, respectively). (c) H&E-stained section showing the whole 4-day-old ovary imaged in (a). (d and f) Enlarged views of the dotted square areas in (a)-3 and (b)-2, respectively. (e and g) H&E-stained tissue sections of the same tissue used for (d) and (f). (h and i) En-face images at depth Z from the tissue surface of mouse ovaries at 20–24 weeks of age (see S5 Video in S1 File). All scale bars = 100 µm.

Fig 4 shows OCM images of mouse ovaries at 4 days (a), 14 days (b), and 20–24 weeks (h, i) of age, reaching a depth of 300 µm. In 4-day-old mouse ovaries, primordial follicles with diameters of approximately 20–40 µm appeared as dark, circular shadows and were localized relatively close to the tissue surface. With increasing depth, primary follicles were also occasionally observed (Fig 4(a)-1, −2, S3 Video in S1 File). For example, Fig 4(d), an enlarged view of the yellow dashed box in Fig 4(a)-3, shows primary follicles surrounded by a single layer of granulosa cells (yellow arrows). In the H&E-stained image of the same ovarian tissue, primary follicles covered by a single layer of granulosa cells (yellow arrows) were also observed (Fig 4(e)), supporting the interpretation of the OCM structures. The density and localization of primordial and primary follicles varied among individuals, even at the same depth.

**Fig 5:**
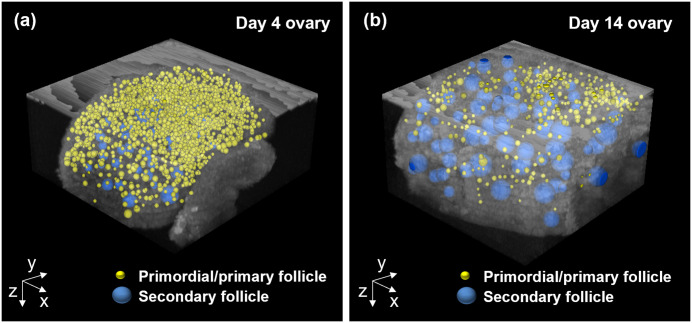
Three-dimensional distribution of follicles in mouse ovaries at (a) 4 days and (b) 14 days of age. Yellow and blue spheres indicate primordial/primary follicles and secondary follicles, respectively (see S6 and S7 Videos in S1 File).

**Fig 6:**
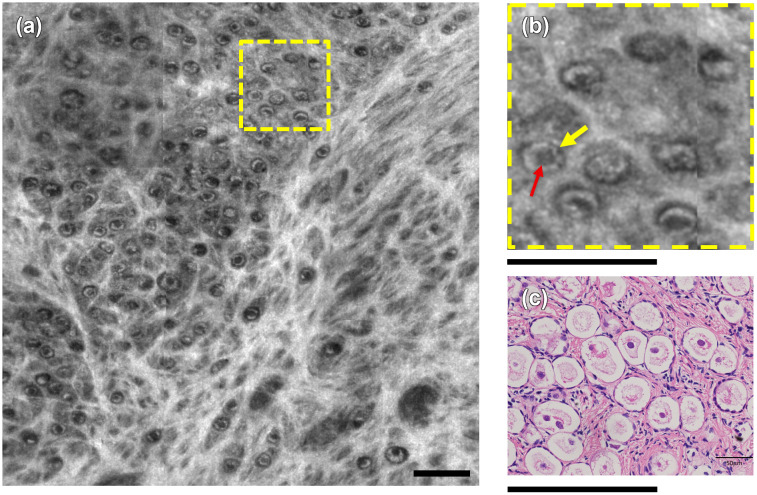
(a) Frontal OCM image at a depth of 120 µm from the ovarian surface of a 15-year-old female patient with acute myeloid leukemia. A cluster of primordial/primary follicles is visible in the upper-left cortex, and primordial follicles are distributed in the lower-right ovarian stroma, which is rich in fibroblast-like spindle cells. (b) Magnified view of the yellow dotted rectangle in (a), with yellow and red arrows indicating oocytes and their nuclei, respectively. No capsule formation is observed around the primordial/primary follicles. (c) H&E-stained section of the same tissue. The circular structures that clearly show cellular and nuclear-like structures are considered to be primordial/primary follicles. Scale bar = 200 µm.

In 14-day-old mouse ovaries, primordial and primary follicle counts were decreased compared to those observed on day 4 and, as shown in Fig 4(b)-1 and −2, were localized within ~100 µm of the tissue surface. Beyond a 100 µm depth, secondary follicles and cyst-like follicles (100–300 µm in diameter) dominated the tissue, with larger follicles concentrated in the central regions (Fig 4(b)-3 to −6, S4 Video in S1 File). In Fig 4(f), which shows an enlarged view of the red-framed area in Fig 4(b)-2, the oocytes (red arrows) and their nuclei (blue arrows) of the secondary follicles—and the thickened granulosa cells (yellow arrows)—were clearly visualized, corresponding to the H&E image of the same tissue shown in Fig 4(g).

In the ovaries of 20- to 24-week-old mice shown in Fig 4(h), follicles ≥600 µm in diameter were observed. In addition to secondary follicle structures, the oocyte (red arrow), cumulus cells (yellow arrow), and follicular cavity (blue arrow) were clearly visualized (Fig 4(h)-4, S5 Video in S1 File). In addition, areas with almost no follicles were identified, as shown in Fig 4(i).

Fig 5 shows the 3D distribution of follicles based on the OCM images shown in Fig 4(a) and 4(b). The slice interval of the OCM images was 3.75 µm, and follicles overlapping multiple slices were counted as one. Primordial/primary follicles and secondary follicles were continuously observed in approximately 5–13 and 14–50 layers, respectively. In 4-day-old mouse ovaries, primordial/primary follicles were predominantly located in the peripheral regions of the ovary, whereas secondary follicles were distributed in the central region, as shown in the 3D images (Fig 5(a), S6 Video in S1 File). Similarly, in 14-day-old mouse ovaries, primordial and primary follicle density was lower than in 4-day-old ovaries, with follicles scattered throughout the peripheral regions (Fig 5(b), S7 Video in S1 File).

### 3.4. Visualization of human follicular microstructures

Fig 6(a) shows an en-face OCM image of ovarian tissue from a 15-year-old AML patient at a depth of 120 μm from the surface. A group of primordial follicles was observed in the parenchymal tissue (upper-left half) and distributed in the spindle-shaped interstitial tissue (lower-right half). In Fig 6(b), which is an enlarged view of the yellow dashed box in Fig 6(a), the primordial follicles in the OCM images were clearly visualized, including the oocytes (yellow arrows) and their nuclei (red arrows). The observed structures were morphologically similar to those observed in the H&E-stained image of the same ovarian tissue presented in Fig 6(c). The theca cell layer surrounding the primordial and primary follicles was not discernible in the H&E-stained images. In the OCM images, mouse primordial follicles appeared as dark shadows, whereas human primordial follicles resembled the structures observed in H&E-stained sections. The mouse ovaries were imaged under sustained physiological conditions postovariectomy, whereas the human ovaries were observed postcryopreservation. The optical properties of the follicles, specifically differences in tissue or cell water content, refractive index, and scattering coefficient, may have caused differences in the OCM images.

**Fig 7:**
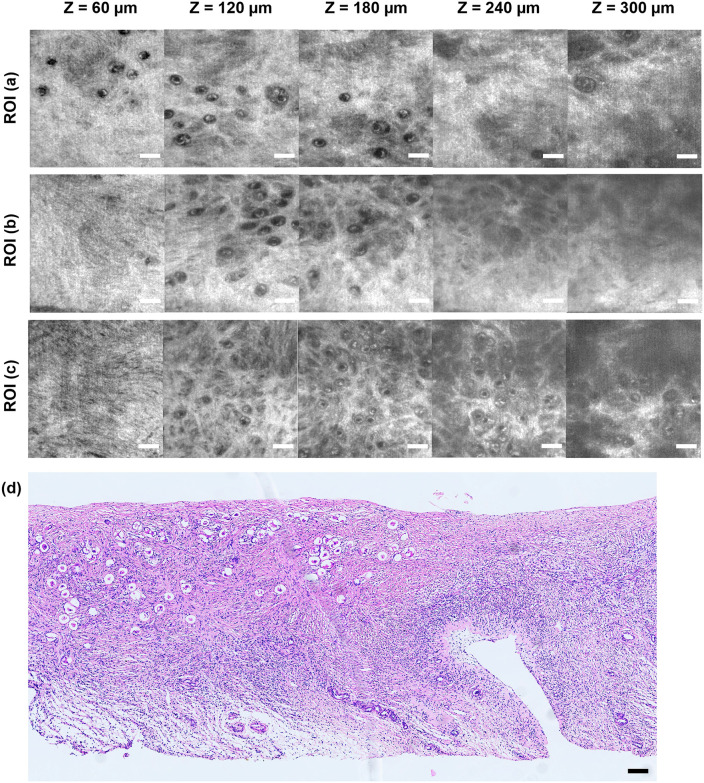
In the same ovary as Fig 6, an uneven distribution of primordial follicles is observed across regions of interest (a)–(c) and at depths of 60–300 µm. (d) A low-magnification, H&E-stained image of the same ovarian tissue similarly demonstrates a region-dependent distribution of follicles. Scale bar = 100 µm (see S8 Video in S1 File).

Fig 7 presents OCM images from the surface to a depth of 300 μm at different regions of interest (ROIs (a)–(c)) in ovarian tissue fragments from the patient with AML (see S8 Video in S1 File). The presence and apparent size of follicles varied with imaging depth and ROI. For example, in ROI (a), follicles were observed from the surface to a depth of 180 μm, whereas they were mainly observed at depths of 120–180 μm in ROI (b). Conversely, in ROI (c), follicles were observed at depths of 120–300 μm. The corresponding H&E-stained cross-sectional image presented in Fig 7(d) also contained areas with multiple follicles alongside regions with few or no identifiable follicles. These observations indicate spatial heterogeneity in the examined AML specimen. Similarly, in ovarian tissue from the patient with ALL, follicles were visualized over a depth range exceeding 500 μm, and their localization appeared spatially nonuniform (see S9 Video in S1 File).

## 4. Discussion

### 4.1. Visualization of follicular microstructure

Visualizing the fine structure of follicles may help distinguish follicular maturation stages, and information on follicle localization may contribute to future ovarian tissue assessment. Because OCT contrast arises from backscattered light generated by refractive index heterogeneity, refractive index differences among ovarian tissue components are important. In the ovarian cortex, the relatively large refractive index difference between the collagen-rich stroma (refractive index of approximately 1.43–1.47) [53,54] and follicular fluid, which has a refractive index similar to water (approximately 1.33–1.35) [55], enables clear delineation of follicular boundaries. In addition, the local refractive index heterogeneity within structures, such as the zona pellucida and granulosa cell layer may contribute to scattering contrast, thereby facilitating visualization of the intrafollicular architecture. In contrast, as shown in Fig 4(a), primordial follicles are often visualized as low-signal circular structures against the surrounding collagen-rich, highly scattering stromal tissue. This is likely because their interiors exhibit limited refractive index heterogeneity and lack a sufficiently complex microstructural organization to generate strong backscattered signals.

**Fig 8:**
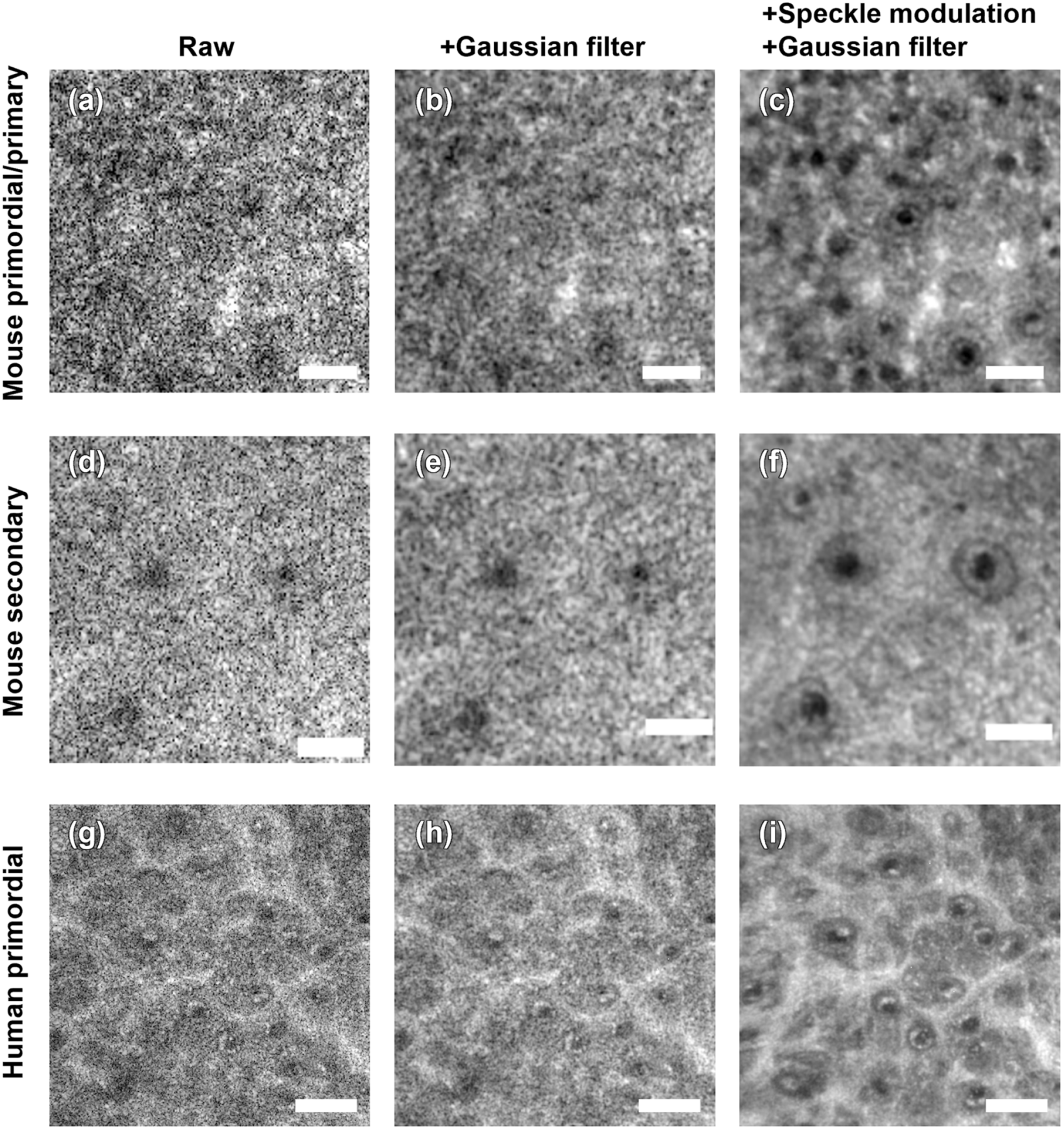
Typical examples of noise reduction using speckle modulation to visualize the fine structure of follicles. Raw OCM images containing speckles of mouse ovaries of (a) mouse primordial/primary follicles, (d) secondary follicles, and (g) human ovarian tissue. (b, e, h) Images processed with a Gaussian filter applied to the original images, and (c, f, i) effects of speckle reduction processing using a diffusion plate. Scale bars: 50 µm for mouse ovary images (a–f) and 100 µm for human ovary images (g–i).

Previous OCT-based follicle imaging was limited by insufficient spatial resolution and inadequate noise removal, making it difficult to distinguish between primordial and primary follicles. As shown in Fig 4(d) and S3 Video in S1 File, we visualized a single layer of granulosa cells in the primary follicle. Speckle modulation was key to visualizing the follicular microstructure. As shown in Fig 8, the fine structures of mouse primordial follicles (a, b) and secondary follicles (d, e)—as well as human primordial follicles (g, h)—were not visible in the original or Gaussian-filtered images. However, they were visualized clearly only when speckle modulation and Gaussian filtering were combined (c, f, i). As described in the Materials and Methods section, applying speckle modulation requires acquiring 20 volumetric datasets via laser irradiation through a scattering plate—a bottleneck that limits acquisition speed.

Mouse ovaries have a less distinct cortical–medullary boundary, with follicles generally distributed throughout the tissue, whereas human ovaries have a distinct follicle-rich cortical layer and a thicker, more fibrous cortex [56–58]. These structural differences may influence optical scattering, as human ovarian tissue has been reported to exhibit an optical scattering coefficient of 2.41 mm^−1^ [59,60]. Despite these structural and optical differences, follicular microstructures were visualized in both mouse and human ovarian tissues to depths of approximately 300–500 μm. The observed differences in imaging depth suggest that interindividual variability may also influence imaging penetration depth. Visualizing follicular microstructures in human ovarian tissue at depths comparable to those achieved in mouse ovarian tissue is meaningful because observations feasible in animal models are not always readily achievable in human tissues.

However, this depth remains insufficient because primordial and primary follicles can exist at depths of up to approximately 1 mm from the ovarian surface. Therefore, further expansion of the imaging depth remains desirable for a more comprehensive assessment of follicle-containing regions within ovarian tissue. Lasers operating in the 1,700-nm wavelength band are suitable for deeper tissue imaging because of their high tissue penetration [61–63]. Although shifting to longer wavelengths can reduce spatial resolution, combining long-wavelength imaging with speckle modulation could enable deeper visualization while maintaining sufficient spatial resolution for follicular microstructure assessment. In addition to signal attenuation, the major limitations of deep OCT imaging include an increase in multiply scattered light, which contributes to background noise and degrades image contrast. One reported approach to suppress these deep multiply scattered components is multifocus averaging, in which volumetric datasets are acquired at different focal positions, computationally refocused, and then complex-averaged [64]. More recently, reflection matrix-based computational optical approaches have been reported to compensate for wavefront distortions caused by forward multiple scattering, enabling approximately 5-fold deeper high-resolution optical imaging beyond conventional limits [65]. Although direct integration of such methods into conventional swept-source OCT systems remains challenging, computational optical approaches may offer a potential route for improving imaging penetration depth.

### 4.2. Spatial variation in follicle localization in the examined human ovarian tissues

Reliable follicle localization may provide useful information for ovarian tissue assessment because preserving tissue with few or no follicles might offer limited reproductive benefit despite the burden of tissue collection, processing, storage, and possible transplantation. In the ovarian tissue from the patient with AML, follicles exhibited a spatially nonuniform distribution. Specifically, primordial follicles were observed in both parenchymal and interstitial regions, and the distribution of follicles differed according to the lateral position and imaging depth. The HE-stained sections were used only as supportive histological observations, and they were not intended to provide rigorous validation or one-to-one co-registration with the volumetric OCM findings. In addition, the ovarian tissue from the patient with ALL contained a relatively large number of follicles, but their distribution was not uniform across the lateral plane or across different depths. Because the present human data were obtained from only two patients with leukemia, they cannot support general biological conclusions regarding human ovarian follicle distribution. These observations suggest that spatially nonuniform follicle localization should be assessed using systematic, reproducible, and eventually automated analytical approaches. A large amount of disease-specific data will be required to clarify the relationship between follicle localization in human ovarian tissue and ovarian reserve. One challenge in assessing follicle localization from OCT images is that manual evaluation by clinicians is impractical, highlighting the need for automated and observer-independent analysis. Machine learning–based medical image analysis can enable reproducible feature extraction and efficient analysis of large datasets [66,67]. Hassan et al. demonstrated that deep learning–based object detection models can automatically identify and quantitatively assess ovarian follicles and corpora lutea from histological images with high accuracy [68]. Saito et al. reported the feasibility of automated follicle detection in ovarian OCT images using a convolutional neural network-based approach [69]. Accurate AI-based feature extraction requires access to fine structural information; therefore, visualization of follicular microstructures and future automated assessment of follicle localization could represent complementary components of ovarian tissue assessment.

### 4.3. Imaging speed and technical challenges

In general, culturing oocytes within the ovary and maintaining their viability are challenging and require careful attention to temperature, gas partial pressures (such as 5–6% O₂ and CO₂), and shielding, as oocytes are light sensitive. Therefore, maintaining follicular viability while rapidly assessing follicle-containing regions in human ovarian tissue requires faster OCM image acquisition. Acquiring a volumetric dataset with a 666 µm lateral width and a 75 µm depth required approximately 3 min. With the high-numerical aperture objective, imaging a single region to a depth of 500 µm would require approximately 15 focal planes and an acquisition time of approximately 1 h under the current conditions.

Implementing machine-learning algorithms offers a promising approach to reducing the number of required data acquisitions. Because object detection accuracy depends on training images with clearly identified follicular structures, machine-learning algorithms trained on follicle images accurately identified via speckle modulation could enable high-precision follicle detection, even from low-quality images with limited averaging.

Using a high-speed light source is another effective way to reduce acquisition time. This study employed a 20 kHz source; however, recent reports have demonstrated light sources operating at 325 kHz [70], which could reduce image acquisition time in future setups.

### 4.4. Assessment of possible risks and safety

OTC is currently considered a promising FP strategy for children and adolescents diagnosed with cancer who face chemotherapy or radiation therapy that may impair ovarian reserve [71]. OTC has been clinically applied in Europe and the United States since the late 1990s. With growing reports of live births, it is no longer considered an experimental procedure in these regions [72,73]. For CAYA cancer patients who cannot delay treatment, OTC remains the only FP option. Consequently, clinical research on its safety and efficacy should proceed in parallel with the development of follicle visualization technology. A previous study demonstrated that in vitro fertilization using OCT-imaged mouse ovaries did not reduce oocyte yield, fertilization rate, or blastocyst formation compared to controls, and no increase in congenital anomalies was observed in the offspring [32]. However, the potential risk of ovarian follicle damage associated with the laser parameters necessary for follicle visualization, such as wavelength, irradiance, exposure time, total energy delivered, and heat generation, requires rigorous evaluation. Moreover, given the interspecies differences in ovarian structure, follicular distribution, and optical properties between mice and humans, stepwise safety validation, including studies in non-human primate models, will be crucial prior to clinical translation. Increasing irradiation energy or scan counts improves image quality but raises the risk of tissue damage. Defining the damage threshold of oocytes to laser exposure using primate and human ovarian tissue is essential to ensure safe transplantation.

Furthermore, in patients diagnosed with leukemia (the most common childhood cancer), neuroblastoma, Burkitt lymphoma, or ovarian malignancies, frozen-preserved ovarian tissue may carry a risk of minimal residual disease (MRD), potentially precluding transplantation. OCT has been explored for tumor detection [74–79]. Consequently, a future OCT-based technique capable of identifying follicle-containing regions while simultaneously detecting MRD lesions may benefit patients for whom OTT is not feasible.

## 5. Conclusion

This study demonstrates that speckle-modulated OCM enables noninvasive visualization of the follicular microstructure in human ovarian tissue at depths beyond the typical range of high-resolution FF-OCT. Although primordial, primary, and secondary follicles were distinguishable in mouse ovarian tissues, visualization of human ovarian tissues at comparable depths suggests the feasibility of extending this approach beyond animal models. With further optimization of imaging speed, extension of the imaging penetration depth, and additional safety validation for laser irradiation, this approach may provide a technical basis for future studies on ovarian tissue assessment in fertility preservation for patients with CAYA-generation cancer.

## Supporting information

S1 File S1 Video: Longitudinal section (B-scan) imaging of an ovarian tissue phantom. S2 Video: Three-dimensional distribution of glass beads in ovarian tissue phantom. S3 Video: Imaging of primordial follicles in a 4-day-old mouse ovary. S4 Video: Imaging of secondary follicles in a 14-day-old mouse ovary. S5 Video: Visualization of mature secondary follicles with follicular cavities. S6 Video: Three-dimensional distribution of follicles in a 4-day-old mouse ovary. S7 Video: Three-dimensional distribution of follicles in a 14-day-old mouse ovary. S8 Video: Imaging of follicles in ovarian tissue from a patient with acute myeloid leukemia. S9 Video: Imaging of follicles in ovarian tissue from a patient with acute lymphoblastic leukemia.
(ZIP)
